# Table Grapes during Postharvest Storage: A Review of the Mechanisms Implicated in the Beneficial Effects of Treatments Applied for Quality Retention

**DOI:** 10.3390/ijms21239320

**Published:** 2020-12-07

**Authors:** Irene Romero, Maria Vazquez-Hernandez, Isaac Maestro-Gaitan, Maria Isabel Escribano, Carmen Merodio, Maria Teresa Sanchez-Ballesta

**Affiliations:** Department of Characterization, Quality and Safety, Institute of Food Science, Technology and Nutrition (ICTAN-CSIC), Ciudad Universitaria, E-28040 Madrid, Spain; irene.romero@ictan.csic.es (I.R.); mavahe86@gmail.com (M.V.-H.); isaac.maestrogaitan@gmail.com (I.M.-G.); escribano@ictan.csic.es (M.I.E.); merodio@ictan.csic.es (C.M.)

**Keywords:** table grapes, fruit quality, postharvest, physical treatments, mechanisms, gene expression

## Abstract

Table grape is a fruit with increasing interest due to its attributes and nutritional compounds. During recent years, new cultivars such as those without seeds and with new flavors have reached countries around the world. For this reason, postharvest treatments that retain fruit quality need to be improved. However, little is known to date about the biochemical and molecular mechanisms related with observed quality improvements. This review aims to examine existing literature on the different mechanisms. Special attention will be placed on molecular mechanisms which activate and regulate the different postharvest treatments applied in order to improve table grape quality.

## 1. Introduction

Table grape cultivars (*Vitis vinifera* L. and *V. vinifera* hybrids with *V. labrusca* L. and *V. amurensis* Rupr.) are from the Vitaceae family of deciduous woody perennial plants, being one of the most consumed non-climacteric fruits worldwide. Table grape is a fruit with a relatively low rate of physiological activity which does not ripen further after harvest. Its quality refers to different attributes related to appearance, color, texture, flavor and aroma. Its evolution begins as the “veraison” stage with the onset of ripening, alongside the accumulation of sugars, berry softening, anthocyanin synthesis, the metabolism of organic acids and the accumulation of flavor compounds [1]. Soluble solid content (° Brix) and sugar/acid ratios serve as primary indices of table grape quality and minimum requirements have been specified for each cultivar. Table grape flavor is a complex and important quality since it is a mixture of hundreds of different volatile compounds which are synthesized during ripening. After harvest, table grapes are highly perishable as they are subjected to important water losses as a result of rachis and pedicel desiccation, causing browning, weight loss and berry softening. Moreover, fungal decay, largely caused by the necrotrophic pathogen *Botrytis cinerea* also produces big losses [2]. This fungus has a high growth rate and a strong ability to spread through berries even at low temperatures around 0 °C. As a result of this, its conservation is limited and depends on numerous internal factors such as the structure and consistency of the skin and pulp, and the maturation ratio, as well as external factors, with temperature and relative humidity being the most important.

Consumers’ high acceptance of table grapes is due to their excellent organoleptic and nutritional qualities, and has led to significant growth in their consumption over recent years. According to 2019 data from the International Organization of Vine and Wine (OIV), approximately 36% of total grape production was destined for fresh consumption, with China being the largest consumer, followed by India and the European Union (EU). Table grape production has doubled in the last twenty years. According to USDA data, world production for 2019/20 is projected to be around 23.4 million tons. Although the impact of COVID-19 has been considered within this global forecast of trade of table grapes, the exact impact of the pandemic is still unclear since its duration and effects on the global economy are still uncertain. China is the major producer (9.5 million tons), followed by Turkey and India who both produce 1.9 million tons (data from OIV for 2018). In reference to world exports, Chile, Italy, and the USA are the three largest exporters of table grapes and, together, these three countries account for 40% of the total. Likewise, many developing countries have substantially increased their table grape exports as it is a source of economic growth. This fact, together with increasing societal awareness about the environment and sustainable development, has promoted research in postharvest technologies to uncover ways of reducing the use of agrochemical compounds, whilst still maintaining the quality of fruit during its storage.

Although in recent years different reviews have described technological advances in maintaining the quality of table grapes during postharvest [2,3,4,5], the mechanisms through which they exert their beneficial effects require an in-depth review. Thus, the present work aims to review existing literature on the different potential mechanisms, giving special attention to molecular mechanisms regulated by the postharvest treatments applied in order to improve table grape quality. Although we have made an effort to review the mechanisms behind different postharvest treatments, it is important to highlight that most published studies up until now examining both the effectiveness of treatments and their mechanisms, have used the modification of storage atmosphere composition as the only postharvest treatment.

## 2. Mechanisms Associated with Effectiveness of the Postharvest Treatments Applied to Maintain Table Grape Quality

### 2.1. Effect on the Cell Wall

Dynamic changes in the chemical composition of the cell wall, as well as in the tissue structure during fruit ripening, senescence and postharvest storage may cause variations in the sensory, chemical and physical properties of table grapes. Plant cell walls are complex, interconnected structures of polysaccharides (pectin, cellulose and hemicellulose), cell wall proteins and polyphenols [6]. The cell wall of grape berries acts as a protection against external factors and forms a barrier to prevent the diffusion of different components including aromas and polyphenols compounds [7].

Firmness of table grapes is an essential quality parameter for consumers and excessive softening may lead to postharvest decay or consumer rejection. Fruit softening occurs primarily through modifications to the cell wall as the result of cell wall polymer degradation which is catalyzed by diverse enzymes such as cellulase (CL), polygalacturonase (PG), β-galactosidase (β-GAL), pectate lyase (PL) and xyloglucan endotransglycosylase/hydrolase (XTH) [8,9]. Changes to the cell wall composition and degrading enzyme activity have been reported during grape ripening [10,11]. Moreover, previous studies indicated that berry firmness during ripening involves complex multigene control [12,13]. Thus, Ma et al. [14] reported that the firmness of Red Globe grapes was significantly higher than that of Muscat Hamburg fruit during ripening, with this seemingly related to the differential expression of the *pectinesterase* (*PE*), *PL*, *PG*, *β-GAL*, *galacturonosyltransferase-like*, *wall-associated receptor kinase-like*, *XTH* and *expansins*. However, the process of berry softening during postharvest is not completely understood and most existing works are focused on changes in enzymatic activities and microstructure, so little is known at a molecular level. Thompson seedless grapes with significantly decreased berry texture following prolonged cold storage have been compared with NN107 fruit, which is a new table grape cultivar with higher berry firmness. This comparison showed differences in cell wall metabolism between both cultivars [15]. Thus, the high calcium content, low uronic acid degradation and reduced PG activity observed in NN107 bunches seem to be associated with its firmer grape berry texture phenotype and higher quality during prolonged cold storage. Moreover, the application of high O_2_ levels in Kyoho grapes (*V. vinifera* L. × *V. labrusca* L.) retarded grape berry softening and reduced levels of PG, PE and β-GAL, and CL activities [16]. At a molecular level, it is known that the application of 30 kPa of CO_2_ for 3 days at 20 °C in detached white wine grapes, down-regulates the expression of genes coding for pectin methylesterase and polygalacturonase. These are two pectin-degrading enzymes which are responsible for softening [17]. Moreover, in other fruits such as strawberries, exposure to 30% CO_2_ for 3 h at 25 °C delayed cell wall degradation, maintaining the integrity of the middle lamella and down-regulating genes related to cell wall degradation enzymes [18]. Short-term high CO_2_ treatments have been described as alleviating flesh gelling during the storage of persimmon at low temperature. This occurs by preserving the integrity of cell walls and the plasmalemma [19]. Low temperature-scanning electron microscopy has been used in CO_2_-treated and non-treated table grapes to examine the microstructure of the epidermal and subsequent hypodermal cells of the skin. The most notable outcome being that grapes stored in air showed greater cellular compression and volume loss [20]. Moreover, cell wall-plasma membrane separation was detected in cells of non-treated Autumn Royal grapes after 41 days at 0 °C. However, double application of a short-term high CO_2_ treatment improved table grape quality and maintained the membrane completely fixed to the cell wall until the end of the cold storage period [21].

### 2.2. Effect on the Plasma Membrane

To date, the mechanisms by which plants sense low temperature are not yet fully understood. Multiple primary sensors are thought to be involved in initial cold stress signaling. Each sensor may perceive a specific aspect of cold stress and may be involved in a distinct branch of the signaling pathway [22]. Plants may sense low temperature through changes in membrane fluidity, permeability properties and fatty acid composition due to an increase in the content of polyunsaturated lipids [23,24].

Due to the importance of maintaining cell membrane integrity during postharvest storage of table grapes at low temperature, the effect of different treatments has been analyzed. The conservation of table grapes in controlled atmospheres with high levels of O_2_ improved the quality of the berries, maintaining the membranes by delaying the increase in ion leakage. This is an indicator of reduced membrane integrity that takes place in fruit stored in air [25]. The application of high levels of CO_2_ significantly reduced ion leakage in Cardinal table grapes stored at 0 °C in comparison to non-treated grapes [26]. A similar effect was reported after exogenous treatment of polyamines in table grapes, presumably due to maintaining membrane integrity via the accumulation of unsaturated fatty acids [27]. Likewise, the application of short-term high CO_2_ treatments in Autumn Royal table grapes maintained the integrity of the microstructure of energy-related cell organelles which are essential for metabolic damage repair and cell membrane restoration. Moreover, a gaseous treatment increased the unsaturation of 18-carbon fatty acids, lipid unsaturation ratio and unsaturated fatty acid index in the membrane of polar lipids [21]. Furthermore, it has been suggested that the application of a 2-day CO_2_ treatment in strawberries adjusts fruit metabolism, resulting in α-linolenic acid accumulation which could confer membrane stability during low temperature storage [28].

In consideration of the important role of the membrane and its lipid composition for maintaining fruit quality during postharvest storage, researchers have endeavored to reveal the molecular mechanisms underlying lipid metabolism [29]. However, little information is available in table grapes during postharvest. Phospholipase D, a key enzyme in membrane lipid metabolism, experienced significantly increased enzyme activation, mRNA accumulation and synthesis of new protein during the early stages of heat acclimation. This suggests that phospholipase D may be involved in the heat response of grapes during postharvest [30]. Temperature-induced lipocalin (TIL) is involved in the transport of sterol molecules to the plasma membrane in response to stress conditions, with this increasing membrane fluidity at low temperature [31]. Induction of *TIL* gene expression in citrus fruit stored at low temperature has also been reported. This observation hints at the participation of these genes in promoting tolerance of cold stress [32]. A comparative large-scale transcriptional analysis has been conducted of the response of table grapes to low temperature and high CO_2_ levels. This revealed that the induction of *TIL* and *LTP* gene expression in response to cold storage could be related to the mechanisms activated in non-treated grapes to overcome low temperature storage [26]. LTPs belong to the most functionally important classes of plant proteins as they not only bind lipids, but have also been seen to transfer them between membranes in in vitro experiments [33]. Moreover, LTP stabilization of membranes under stress has been explicitly demonstrated, particularly in the case of cold stress [34].

Another important factor in the study of biomembranes is the specific effect that water exerts on them. Although grapes present a low respiratory rate after harvest and the layer of cuticular wax controls water movement between the epidermal cells and the ambient atmosphere [35], small modifications in the content or state of water can be responsible for very significant changes in the metabolism of grape bunches [36,37]. Likewise, the loss of water equivalent to about 5–10% of fruit fresh weight can render the fruit commercially unacceptable [38]. On the other hand, rachis lacks the thick epidermis and cuticular wax depositions that protect berries against dehydration. Thus, water losses of 2% to 3% are sufficient to make rachis show symptoms of browning [39]. This affects markets in which the condition of rachis grapes, in terms of color and turgor, is a good indicator of postharvest quality. In this sense, it has been observed that storage at 0–1 °C and a relative humidity of 95% is not enough to control water loss from bunches, with this being linked to an increase in rachis browning [40,41,42]. However, postharvest treatments based on altering the composition of storage atmospheres such as MAP, CA and 3-day short-term high CO_2_ treatment reduced water loss and controlled rachis browning during storage [41,43,44].

The degree of water availability determines the properties of membrane lipids and, therefore, can affect the biophysical properties of membranes. Cold storage processes in tulip bulbs were accompanied by conversion of bound water to free water [45]. In line with these results, unfreezable water levels in the different tissues (skin, pulp, seeds and rachis) of Cardinal table grapes measured by differential scanning calorimeter, increased rapidly in response to high CO_2._ In contrast, these water levels remained stable or decreased in grapes stored in the air [36]. Moreover, cold acclimation of vegetative apple buds seems to involve several processes including an increase in the levels of unfreezable water [46]. Furthermore, Blanch et al. [37] indicated that storage of strawberries in air at 0 °C led to a marked decrease in the unfreezable water proportion, an increase in cellular water leakage, cell structure disorganization and water potential. On the other hand, a 3-day CO_2_ treatment led to the maintenance of a similar water status as that found in freshly harvested fruit. Unfreezable water content is associated with membranes, proteins and macromolecules [47]. Goñi et al. [36] indicated that increases in unfreezable water content may, therefore, constitute a sensory parameter that reflects metabolic adaptations in CO_2_-treated tissues due to the alterations caused by storage in air at severely low temperature.

### 2.3. Effect on Oxidative Stress

Apart from the direct effect of low temperature storage on the molecular organization of membrane lipids, the loss of integrity of the membrane itself is boosted by oxidative processes. This is because low-temperature stress increases levels of reactive oxygen species (ROS) (reviewed in [48]). When plants are subjected to environmental stress such as low temperature, the balance between ROS production and detoxification is upset, often resulting in oxidative damage. Plants are protected against the effects of ROS by their complex antioxidant system. This includes antioxidant metabolites such as ascorbate, glutathione, α-tocopherol and β-carotene, alongside antioxidant enzymes such as superoxide dismutase (SOD), catalase (CAT), ascorbate peroxidase (APX) and glutathione reductase (GR) [49,50,51]. In contrast to earlier views, it is becoming increasingly evident that even during stress, ROS production is not necessarily a symptom of cellular dysfunction but might represent a necessary signal in adjusting the cellular machinery to the altered conditions (reviewed in [51]). ROS, especially H_2_O_2_, are proposed to perform multiple functions such as acting as a signal transducer in plant defense against both biotic and abiotic stresses, including low temperature (reviewed in [52]).

It is known that fruit modulate their antioxidant defenses during storage at low temperature or in response to different postharvest treatments. Pavez et al. [53] reported that peach fruit were able to respond to the increased production of ROS induced by extended cold storage. This occurred through the induction of genes encoding CAT, SOD and GR. Moreover, high CO_2_ levels can mitigate the impact of heat on different coffee species (*Coffea arabica* and *Coffea canephora*) by increasing the antioxidant activity of enzymes such as SOD, APX, GR and CAT [54]. In strawberries, there is a marked increase in GR activity associated with the NADPH produced by active malic acid decarboxylation. This may account for the increased levels of glutathione seen in fruit following exposure to 20% CO_2_ [55] Moreover, Yuan et al. [56] observed the induction of different antioxidant enzymes including APX, glutathione S-transferase and oxidoreductase during the storage of Kyoho grapes at 2 °C. In a recent study, it was reported that accumulation of *CAT* and *APX* transcripts in the skin of non-treated grapes from black (Autumn Royal) and white (Superior Seedless) cultivars seems to be activated. This may take place in order to overcome oxidative stress due to cold storage, with this being less noticeable in CO_2_-treated samples [44]. Conversely, Ni et al. [57] reported that gaseous treatment with hydrogen sulfide alleviated postharvest senescence and rotting in Kyoho grapes. This occurred by enhancing the activity of APX and CAT in both grape skin and pulp during storage. A transcriptome analysis showed that the molecular signature of SO_2_ application was highly similar to superoxide stress, evoking a broad metabolic reprogramming in table grape berries. This was consistent with an up-regulation of tolerance and mechanisms, and the associated ROS/redox [58]. However, the beneficial effect of high CO_2_ levels on controlling oxidative stress by modulating the genes implicated in the enzymatic antioxidant system, depended on tissue type and was closely related with browning reductions in rachis grapes, specifically, rather than with a general response in table grapes [26,44].

It is known that when ROS levels exceed the capacity of plants to scavenge, lipid peroxidation in biological membranes increases, thereby affecting the physiological processes of the cell. Malondialdehyde (MDA) is one of the final products of the oxidative modification of lipids. Its content reflects the state and integrity of plant cell membranes and has been extensively used as an indicator of oxidative injury. In this respect, MDA production is clearly implicated in the symptoms of environmentally stressed plants [59]. It is known that the application of postharvest treatments enhances chilling tolerance in fruit and reduce MDA content [60,61,62]. In table grapes, different studies have indicated that storage atmosphere modifications control the MDA content increases activated during cold storage. In this way, 3-day CO_2_ treatments applied either once or twice have delayed postharvest cold-increase in H_2_O_2_ and MDA content in Autumn Royal berries [21]. Moreover, the reduction in rachis browning and MDA content observed in 3-day CO_2_-treated Cardinal bunches was linked to activation of the enzymatic antioxidant system. This was also associated with significant increases in *APX* and *CAT* gene expression in this tissue [41]. Furthermore, postharvest application of nitric oxide gas at low temperature maintained the quality of Munage table grapes reducing the accumulation of O_2_^•−^, H_2_O_2_ and MDA content via the activation of ROS-related genes and antioxidant enzymes [63].

### 2.4. Effect on Phenylpropanoid Metabolism

Phenolic compounds are a group of important secondary plant metabolites which are associated with fruit quality, thus affecting appearance, taste and flavor. Specifically, grapes constitute one of the major sources of phenolic compounds amongst different fruit species [64]. These compounds have received increasing scientific interest due to their antioxidant properties and potential beneficial health effects. Thus, since the postharvest storage of table grapes may have an important impact on the phenolic compounds and enzymes involved in their metabolism, the examination of their biosynthesis and regulation is of special interest. Different studies have indicated that there is no common trend regarding total phenolic content changes, in response to postharvest treatments that improve table grape quality. The total phenolic content of red and white table grapes was found to decrease and stay the same, respectively, following 2 days of storage at 4 °C, increasing thereafter in both cultivars [65]. Postharvest chitosan coating treatments decreased total phenol content during storage at 0 °C and also during shelf-life [66]. However, the application of UV-B and UV-C significantly increased the content of total phenolic compounds during table grape storage at 4 °C [67]. Artés-Hernández et al. [68] reported that white Superior Seedless table grapes stored for 7 days at 0 °C, followed by 4 days at 8 °C under modified atmosphere packaging, did not change their total phenolic content. Further slight decreases were seen during their subsequent shelf-life. In contrast, in the same cultivar of Superior Seedless grapes, application of a 3-day CO_2_ treatment at 0 °C increased phenolic content [44]. However, it is important to note that varietal factors and the stage of ripeness could determine the concentration, distribution and accumulation of polyphenols in grapes [68,69,70]. Further, total phenolic content decreased after 3 days of storage at 0 °C in black table grapes but did not vary in white ones [44].

The oxidative degradation of phenolic compounds catalyzed by polyphenol oxidase (PPO) and peroxidase (POD) enzymes is one of the most important issue to the food industry as it results in the enzymatic browning of fruit and vegetables [71]. It has been proposed that PPO, normally found in the chloroplast, comes into contact with the substrate in the vacuole due to the loss of compartmentalization caused by desiccation [72]. The development of rachis browning during the postharvest storage of table grapes has been associated with PPO activity [73]. Rosales et al. [41] observed that the application of a 3-day CO_2_ treatment at 0 °C reduced the rachis browning index and sharply increased *PPO* gene expression in non-treated bunches. In this sense, the application of hexanal formulation to improve table grape quality suppressed PPO-related browning which maintained rachis freshness [74].

Phenylalanine ammonia lyase (PAL) catalyzes the first reaction in the phenylpropanoid pathway, producing a wide variety of phenolic compounds which include flavonoids, phytoalexins and phenolic esters [75,76]. The activation of phenylpropanoid metabolism may play a role in plant defense against pathogens, as well as in the response to abiotic stresses [77,78]. Furthermore, modification of the phenylpropanoid biosynthesis pathway becomes is eased by the induction of mutations or genetic engineering, resulting in a shift in physical as well as metabolic functioning of the pathway [78]. Of the polyphenol compounds derived from the phenylpropanoid pathway, stilbenes represent defense biomarkers, because they occur as phytoalexins whose production is induced dynamically in response to biotic or abiotic stress. Stilbene synthase (STS) catalyzes, in a single reaction, the formation of simple monomeric stilbenes (e.g., resveratrol, pinosylvin and piceatannol) from coenzyme A-esters of cinnamic acid derivatives and three malonyl-CoA units [79]. The remaining stilbenes are formed as a result of glycosylation (piceids) and hydroxylation (piceatannol and its glycoside, astringin) reactions, and the formation of dimers and trimers (viniferins).

Vanozzi et al. [80] characterized the *STS* multigenic family from *V. vinifera* into 48 different members. *STS* genes have been transformed into different plants in order to improve resistance against fungal pathogens and other abiotic stresses [81,82,83], whilst also increasing either resveratrol accumulation [81,84] or piceid accumulation [85]. Moreover, resveratrol content increased in table grapes in response to the application of different postharvest treatments such as UV-B and UV-C irradiation [86], the combination of UV-C and chitosan coatings [87] and ozone treatment [88]. In relation to the transcriptional regulation of *STS* genes in table grapes, induced expression linked to increases in the accumulation of resveratrol has been reported following the application of red and blue light-emitting diodes [89] and ultrasonication [90]. Moreover, Maoz et al. [91] analyzed the expression of six *STS* genes in white Superior Seedless grapes treated for 6 weeks with 15% CO_2_ and 5% O_2_ at low temperatures. They observed increased induction at both low temperature or high CO_2_ levels, together with the accumulation of six viniferins and an unidentified resveratrol dimer. A recent study reported that *STSs* gene expression and the accumulation of stilbene compounds following the application of short-term CO_2_ treatment at low temperature was cultivar dependent [92]. These authors reported that a 3-day high CO_2_ treatment activated the expression of four *STSs*, whilst also activating stilbenes in the white Dominga cultivar. However, accumulation of these compounds increased in non-treated Red Globe grapes at 0 °C, with this also seeming to be activated by STS expression. Moreover, prolonged storage at 0 °C of non-treated samples of Cardinal grapes, another red cultivar, led to increased *trans*-resveratrol levels but only at the end of storage [40].

Flavonoids are sensitive to different biotic and abiotic stress as part of the plant adaptation mechanisms to the environment [93]. Furthermore, it is known that the quantitative and qualitative composition of flavonoids depends on the table grape cultivar, whilst the influence of postharvest storage on their contents does not follow a regular pattern [91,94,95,96]. With regards to transcriptional regulation, little is known about whether the expression of branch-specific flavonoid genes is regulated by different postharvest treatments in table grapes. This is because most published works have been restricted to a small number of genes, most of which belong to the common pathway [97,98]. UV-B and UV-C treatments activated the expression of genes specific to flavanol formation, such as *ANR (anthocyanidin reductase)* and *LAR* (*leucoanthocyanidin reductase*), in Summer Black table grapes [67]. Likewise, the application of melatonin to Kyoho grapes combined with storage at 22 °C for 3 days significantly enhanced the biosynthesis of flavanol components, whilst also increasing expression of *flavanol synthase* (*FLS*) and *flavonoid 3′,5′-methyltransferase* (*AOMT*). In contrast, *chalcone isomerase* (*CHI*), *flavanone 3-hydroxylase* (*F3H*), *dihydroflavonol 4-reductase* (*DFR*), *leucoanthocyanin dioxygenase* (*LDOX*) and *ANR* accumulation was inhibited [99]. Likewise, *PAL* and *LAR2* expression seems to be a regulating factor of catechin accumulation in CO_2_ table grapes [96]. Moreover, the grape genotype seems to be an important factor to be considered for the modulation of common flavonoid genes, following short-term CO_2_ treatments applied at low temperature [96,97]. However, SO_2_ application for 21 days strongly down-regulated *LAR* and *ANR* transcript accumulation [14].

With regards to anthocyanins, it is known that low temperature postharvest storage activates modulation in different fruits including table grapes. Furthermore, various studies have shown that treating harvested fruit with high levels of CO_2_, for instance by controlling the atmosphere or modified atmosphere packaging, inhibits the increase of anthocyanin by affecting its biosynthesis, degradation, or both (reviewed by [100]). However, whilst a decrease in the content of all identified anthocyanins has been recorded in black Autumn Royal table grapes stored at 0 °C, a dual short-term CO_2_ treatment was seen to maintain levels after 28 days at 0 °C similar to freshly harvested fruit, which was correlated with the expression of *CHS*, *LDOX* and *UDP-glucose: flavonoid 3-O-glucosyltransferase* (*UFGT*) genes [96]. Moreover, the application of light irradiation at 15–25 °C as a postharvest technique for stable production of well colored grapes, also increased anthocyanin levels. This was linked with greater induction of anthocyanin biosynthesis-related genes including *CHS*, *LDOX* and *UFGT,* and two *MYB*-related transcription factors [101]. Likewise, low nocturnal temperatures increased anthocyanin synthesis in black grape berries through the regulation of *PAL* activity and *CHS* gene expression [102].

### 2.5. Molecular Basis of the Response of Table Grapes to Postharvest Treatments

The sequencing of the *V. vinifera* cultivar Pinot Noir resulted in the first genome of a woody crop species [103,104]. Numerous assemblies and annotations of the PN40024 reference genome [103] have been performed, with the latest version (12 × v. 2 assembly and VCost.v. 3 annotation) improving contig coverage and orientation by 14% over the previous assembly (12 × v. 0) and annotation (v. 1) [105].

The generation of transcriptome data together with functional analysis of identified candidate regulatory genes, represent powerful tools for unraveling the molecular mechanisms underlying the response of table grapes to postharvest treatments. Thus, high-throughput transcriptomic analysis via microarrays has become a key tool for understanding these molecular changes. Becatti et al. [17] used the Array-Ready Oligo Set™ containing 14,562 probes to examine detached wine grapes following treatment with 30 kPa of CO_2_ for 3 days at 20 °C. They found this treatment to be effective at altering general metabolism, with functional categories related to protein and hormone metabolism, transport and stress being more highly represented in both skin and pulp tissues. A transcriptome analysis using the GeneChip^®^ *V. vinifera* genome array from Affymetrix and containing probe sets for approximately 16,000 genes, suggested that SO_2_ application has similar effects to superoxide stress. This includes the down-regulation of *WRKY* and *bHLH* transcription factors, which are strongly up-regulated by other ROS stresses. Similarities were also seen with profiles associated with ROS, other than superoxide, including the up-regulation of *ERFs* transcription factors [58]. Moreover, a custom Affymetrix GrapeGen GeneChip^®^ [106] containing 23,096 probe sets corresponding to 18,711 non-redundant transcripts was used to examine changes in the transcriptome of Cardinal table grape skin at different stages of maturity. Following exposure to high levels of CO_2_ and 0 °C for 3 days, it was seen that the gaseous treatment seems to be an active process requiring the activation of different transcription factors, including *ethylene response factors* (*ERFs*), *WRKYs* and *MYBs* [26].

In taking a closer look at the role of transcription factors it is important to note that most studies in *V. vinifera* have been performed on grapevine seedlings or during grape ripening [107,108,109,110,111,112] with little being known about their role postharvest. A short-term high CO_2_ treatment has been observed to activate the expression of transcription factors belonging to the APETALA2/ethylene response factor (AP2/ERF) family. These factors include four *ERFs* [113] and three *CBF/DREBs* [42], in addition to nine *WRKYs* [114] seen in red and black table grapes. These transcription factors evidenced “switch mode” expression patterns, with induction taking place at the end of gaseous treatment followed by a rapid decline. It is important to note that similar results were observed as a response of fruit to low temperature during postharvest storage [115,116]. This suggests that this mode of modulation is a factor in turning on transcription factors and, consequently, activating or repressing target genes. This may be critical for fruit protection from stress damage during postharvest. However, Maoz et al. [91] showed that storage of white Superior Seedless grapes for 6 weeks, either at 0 °C in air or with 15 kPa CO_2_, did not activate the expression of *ERFs* and *CBFs* in comparison to freshly harvested fruit. However, it is difficult to compare results because the gaseous treatments applied were different, with Maoz et al. [91] applying a continuous gaseous treatment for 6 weeks and Romero et al. [113] opting for a 3-day short-term treatment. Further, nine *WRKYs* genes were also induced in 3-day CO_2_-treated Autumn Royal table grapes stored at 0 °C [114].

Moreover, different studies have shown that CBF/DREB [42], ERF [113] and WRKY [114] recombinant proteins from table grapes bind in vitro to the regulatory regions of their downstream target genes, such as pathogenesis related proteins (PRs) and dehydrins. Multiple PR isoforms belonging to the chitinase and *β*-1,3-glucanase family have also been reported in grapes [117,118], but available data concerning their expression in grape berries are limited. Increased levels of *chitinase* and *glucanase* transcripts and activity have been reported in grapevines infected with *B. cinerea* (reviewed in [119]). Busam et al. [117] reported that the expression of a class III chitinase gene might serve as a marker for systemic acquired resistance in grapevine leaves. Likewise, the introduction of both β-1,3-glucanase and chitinase genes into transgenic Crimson Seedless lines confers markedly improved resistance to downy mildew [120]. Furthermore, Crimson Seedless lines expressing cisgenic thaumatin-like proteins from *V. vinifera* Chardonnay have displayed resistance to powdery mildew [121].

It has been reported that PRs seem to have a protective role in table grapes postharvest. Thus, overexpression of chitinase and *β*-1,3-glucanase proteins from table grapes in *Escherichia coli* has been shown to boost in vitro cryoprotective activity and antifungal activity [116,117]. Application of SO_2_ or O_3_ caused delayed decay by *B. cinerea* in Redglobe and Sugraone grape cultivars, however, whilst gene expression of chitinase and *β*-1,3-glucanase was increased in treated Redglobe grapes, neither treatment enhanced expression of these PR genes in Sugraone grapes. This may partly explain why this cultivar is more susceptible to decay [122]. Likewise, Wang et al. [123] recently observed that the reduction of grey mold decay caused by *B. cinerea* in Kyoho grape berries treated with β-aminobutyric acid could be attributed to systemic acquired resistance (SAR) defense. This is because a significant increase in the transcript levels of *VvPR1* and endogenous salicylic acid content was observed following β-aminobutyric acid elicitation.

With regards to dehydrins in *V. vinifera*, it has been reported that four of five examined genes showed alternative splicing, leading to the retention of an intron and giving rise to truncated proteins [124,125,126,127]. Fernandez-Caballero et al. [125] described intron retention in *VviDHN1a* transcripts in response to both cold storage and a 3-day CO_2_ treatment at 0 °C, applied in the skin, pulp, seeds and rachis of Cardinal table grapes. This was the first time that this scenario was described in relation to a gaseous treatment in plants. The outcomes have been recently confirmed in another table grape cultivar, Autumn Royal. In this case, Vazquez-Hernandez et al. [42] observed that unspliced forms showed a higher extent of regulation than spliced forms after the application of high levels of CO_2_ at 0 °C. Although the functionality of unspliced DHNs is not well known, it has been observed that recombinant unspliced DHN1a variant of *V. vinifera* cv. Cardinal, slightly interacts with DNA. However, a potential role of spliced DHN forms has been indicated in protection against freezing and dehydration, as well as inhibiting *B. cinerea* growth [128].

Transcriptome sequencing using next-generation sequencing technologies has been increasingly carried out in model and non-model plants for gene detection and marker development. Compared with traditional laboratory methods, RNA-Seq is a high throughput technology, overcoming the weakness of microarrays in exploring unknown genes. To date, RNA-seq technology has been widely used to detect gene expression in different tissues such as berries, flowers and leaves, and in response to different environmental stresses [129,130,131,132,133,134,135,136]. Interestingly, although most grapevine transcriptome research has focused on wine grape cultivars due to their high economic demand, different studies have been performed over recent years in table grapes. Some of these examined global transcriptome changes during table grape ripening [137,138] and in response to preharvest treatments [139,140]. Using RNA-seq and LC–MS/MS techniques, Zhang et al. [141] analyzed the resistance of Kyoho and Shine Muscat grapes to *B. cinerea* infection following chitosan treatment. They found that this treatment significantly regulated the emergence of fungal diseases, plant hormone biosynthesis, signal transduction and secondary metabolism. Moreover, an RNA-seq analysis revealed an enrichment of the genes involved in pyruvate metabolism and phenylpropanoid pathways in Superior Seedless table grapes stored at low-temperatures for 6 weeks under elevated CO_2_ levels [91].

The advent of genome editing now offers revolutionary tools to edit and completely knock out susceptibility genes in many crops, whilst preserving their cultivar and clonal genetic backgrounds. The CRISPR/Cas9 system has been utilized successfully for mutagenesis in a variety of organisms, including plants such as Arabidopsis, sorghum, rice, tomato, maize, wheat, potato, poplar, orange, liverwort, petunia, and cucumber (reviewed in [142]). In addition, it has been reported successful targeted mutagenesis in grape suspension cells or protoplast using the CRISPR/Cas9 system [143,144,145]. CRISPR/Cas9 technology was recently used to edit powdery mildew and downy mildew susceptibility genes, such as *DOWNY MILDEW RESISTANCE 6* and *Mildew Locus O*, in different grapevine clones [146]. Moreover, CRISPR knockout of *VvWRKY52* in grape increased resistance to *B. cinerea* [147]. Likewise, in a recent study, Li et al. [148] engineered loss-of-function mutations in the *VvPR4b* gene from the cultivar Thompson Seedless using the CRISPR/Cas9 system. Results showed that *VvPR4b* knockout lines increased susceptibility to pathogen infection, which was accompanied by a reduced accumulation of ROS around stomata. These recent works confirm that grape is becoming a pioneering fruit crop in the area of transgene-free genome editing, however, further work is needed in order to modify table grapes and improve their quality during postharvest storage.

## 3. Conclusions and Future Perspectives

**Figure 1:**
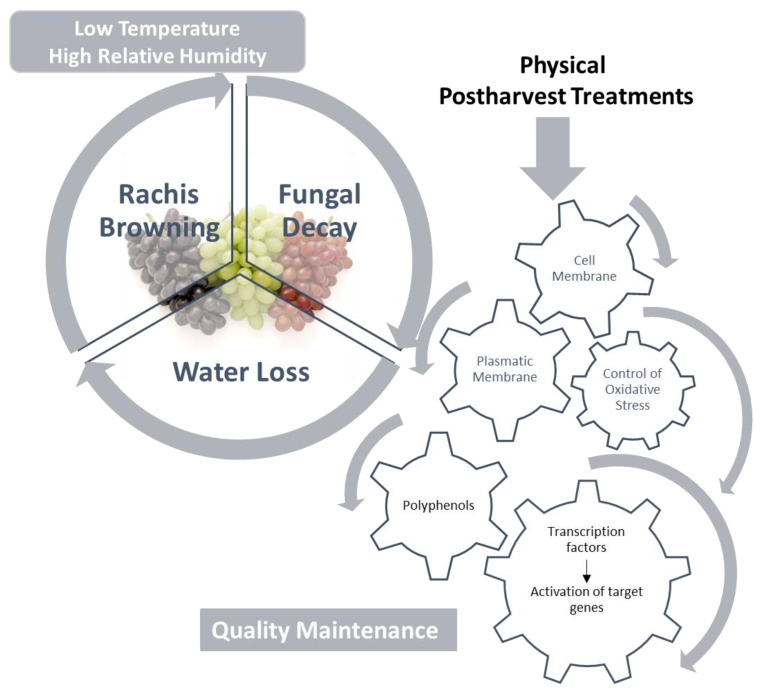
A scheme summarizing responses of table grapes to physical treatments during postharvest storage.

In the present day, different postharvest technologies are employed in order to improve table grape quality during postharvest storage. Consumer reluctance regarding the chemical treatment of agricultural products has promoted the use of physical treatments. These consist mainly of the control of the gaseous composition surrounding bunches during storage at temperatures around 0 °C. For this reason, most currently existing published works analyze the mechanisms underlying the beneficial effects of postharvest treatments with regards to gaseous treatments. Such treatments exert effects to preserve grape firmness by maintaining the cell wall through the regulation of genes coding for cell wall degrading enzymes. Moreover, various postharvest treatments served to maintain membrane integrity, thus, water status seems to be an important factor to consider when analyzing different postharvest treatments. However, no common trends are seen in relation to the modulation of the antioxidant system through the application of postharvest treatments. Indeed, effects typically depend on the type of tissue analyzed. In contrast, most postharvest treatments served to control lipid peroxidation by reducing MDA content, with this being activated during cold storage. The phenylpropanoid pathway has been extensively studied due to its implications for the health-promoting properties of table grapes. These studies show that various treatments promote increased levels of the final compounds such as anthocyanins, stilbenes and flavanols, through the modulation of gene expression. However, some of these responses were cultivar-dependent. Evolution of molecular tools, such as microarrays and RNA-seq, has allowed to uncover the role of different transcription factors on the effectiveness of postharvest treatments applied to table grapes. These transcription factors were not only modulated at a transcriptional level by the different treatments, but there was also evidence of its role in the in vitro activation of target genes such as those coding for PRs and dehydrins (Figure 1).

Finally, although most research using CRISPR/Cas9 gene-editing technology has been focused on improving susceptibility to pathogen infection in grapes, application of this system opens up an interesting research area to achieve better quality modified table grapes. Deepening knowledge around the molecular basis implicated in the maintenance of table grape quality should be a goal of future table grape postharvest research.

## Abbreviations

ANR	Anthocyanidin reductase
AP2/ERF	APETALA2/ethylene response factor
APX	Ascorbate peroxidase
CAT	Catalase
CL	Cellulase
CHI	Chalcone isomerase
DFR	Dihydroflavonol 4-reductase
ERFs	Ethylene response factors
FLS	Flavanol synthase
F3H	Flavanone 3-hydroxylase
AOMT	Flavonoid 3′,5′-methyltransferase
GR	Glutathione reductase
OIV	International Organization of Vine and Wine
LAR	Leucoanthocyanidin reductase
LDOX	Leucoanthocyanin dioxygenase
MDA	Malondialdehyde
PRs	Pathogenesis related proteins
PL	Pectate lyase
PE	Pectinesterase
POD	Peroxidase
PAL	Phenylalanine ammonia lyase
PG	Polygalacturonase
PPO	Polyphenol oxidase
ROS	Reactive oxygen species
° Brix	Soluble, Solid content
STS	Stilbene synthase
SOD	Superoxide dismutase
SAR	Systemic acquired resistance
TIL	Temperature-induced lipocalin
UFGT	UDP-glucose: flavonoid 3-O-glucosyltransferase
XTH	Xyloglucan endotransglycosylase/hydrolase
β-GAL	β-galactosidase
